# Rating Opportunity for Long-Acting Injectable Antipsychotic Initiation Index (ROLIN)

**DOI:** 10.3389/fpsyt.2021.767756

**Published:** 2021-12-07

**Authors:** Petru Ifteni, Paula-Simina Petric, Andreea Teodorescu

**Affiliations:** Faculty of Medicine, Transilvania University of Braşov, Braşov, Romania

**Keywords:** schizophrenia, antipsychotic, treatment, relapse, outcome, long-acting injectable

## Abstract

**Background:** Schizophrenia is a severe psychiatric condition with devastating consequences for the individual's functionality and leading to severe disability. Lack of insight and non-adherence to treatment remain the most important factors in the progression of the disease to chronicity. Despite their proven effectiveness in preventing relapses, reducing morbidity and mortality, long-acting injectable antipsychotics (LAIs) are still underused. One of the causes invoked is the lack of guidelines or protocols for initiating LAIs.

**Objective:** The aim of this article is to present Rating Opportunity for Long-Acting Injectable Antipsychotic Initiation Index (ROLIN), a clinician-rated index that rates the important factors of the disorder across seven items: age, duration of illness, relapses, antipsychotic treatment response, family support, antipsychotic existing formulation and adherence.

**Method:** A retrospective study in which all patients with schizophrenia discharged on oral antipsychotics without LAIs treatment lifetime were evaluated with ROLIN for opportunity for LAIs initiation.

**Results:** Of 225 consecutive patients, 126 patients (56%) had a strong indication for initiating LAI (score between 25 and 35). Kolmogorov-Smirnov test was used for checking the normal distribution of values (95% CI for the mean = 9.5781 to 20.4219; 95% CI for the median = 6.5920 to 24.8161; SD = 9.7907; Coefficient of Skewness = 0.0743; Coefficient of Kurtosis = −1.1377).

**Conclusion:** This paper proposed an instrument designed to improve treatment in schizophrenia using a simple conceptual model which integrates important predictors of good or poor outcomes.

## Introduction

Schizophrenia is a severe psychiatric condition with devastating consequences for the individual's functionality, leading to severe disability (1, 2). In addition, patients with schizophrenia have high levels of cardiovascular comorbidity (3) metabolic diseases (4), sudden death (5) and lower life expectancy (6).

According to a study published in 2016, schizophrenia contributes with more than 13 million life years lived with disability (YLDs) to burden of disease globally, equivalent to 1.7% of total YLDs globally (7).

Treatment non-adherence is the most important factor for the disease's progression. (8–10). Non-adherence has multiple causes related to illness (lack of insight, cognitive impairment, symptoms, etc.), patient (attitude toward medication, stigma, etc.), family (attitude toward symptoms, attitude toward medication and stigma), society (costs, treatment facilities, etc.), antipsychotic treatment (route of administration, number of administrations and side effects) (11).

It is unanimously accepted that sustained antipsychotic treatment is the only proven way to determine remission, maintain it and prevent relapses (12, 13).

Despite their proven effectiveness in preventing relapses, reducing morbidity and mortality, LAIs (long-acting injectable antipsychotics) are still underused (14–16). The main reasons for low utilization are patient-related (fear of needles, stigma, desire not to be under control, etc.) but also clinician- related (costs, personal beliefs or lack of experience) (17, 18). Besides these aspects, clinicians invoke the lack of guidelines or protocols for initiating LAIs. As a result, LAIs initiation is often delayed, or they are used in severe forms of the disease or even in cases that are considered treatment-resistant (19, 20).

Guidelines released by the American Psychiatric Association (APA) or authorities, organization and recommendation of experts from other countries to improve quality of care and treatment outcomes in patients with schizophrenia tend to have general consideration (21–23). There are no initiation guidelines or protocols regarding LAI treatment initiation, but only indications and suggestions from experts (24, 25). Often psychiatrists consider that “it is not yet the time” or “it is too early” or they consider that the costs are too high and the supervision must be more careful, thus delaying initiation.

Developing an instrument for psychiatrists, designed to provide quick guidance on the opportunity to initiate LAI treatment, could improve adherence and consequently the outcomes in schizophrenia.

This paper describes an instrument called Rating Opportunity for Long-Acting Injectable Antipsychotic Initiation Index (ROLIN), designed to improve initiation of LAIs in schizophrenia. ROLIN is a conceptual model, developed by an experienced schizophrenia research group led by Petru Ifteni, M.D., PhD, and professor of psychiatry at Transilvania University of Brasov, Romania, Faculty of Medicine. This clinician-rated instrument is scored using available clinical information for seven domains. Each item is rated on a 3-point scale with 5 points, 3 points, and 1 point. Based on the patient's data, the clinician will choose one value (e.g., age between 18 and 25 will be scored with 5 points) for all seven items. The final score is the sum of all seven items' scores (see Methods; Table 1).

**Table 1:** Rating opportunity for long-acting injectable antipsychotic initiation index (ROLIN).

**DOMAINS**	**Score**
I. Age	
• 18–25 years	5 points
• 26–35 years	3 points
• >35 years	1 point
II. Duration of illness	
• 2–5 years	5 points
• 6–10 years	3 points
• >10 years	1 point
III. Relapse	
• 3 or more relapses	5 points
• 2 relapses	3 points
• 1 relapse	1 point
IV. Response to oral antipsychotic	
• good response/remission	5 points
• partial response	3 points
• poor response/treatment-resistant	1 point
V. Patient social support	
• 2 or more family members	5 points
• 1 family member	3 points
• no family member	1 point
VI. Antipsychotic formulation	
• both oral and LAI	5 points
• only oral	3 points
• clozapine	1 point
VII. Treatment adherence	
• non-adherence	5 points
• partial adherence	3 points
• good adherence	1 point

## Methods

### Study Design and Procedure

We conducted a retrospective study in the Clinical Hospital of Psychiatry and Neurology in Brasov, an academic setting with 150 beds for acute psychiatric patients. The inclusion criteria were: diagnosis of schizophrenia according to Diagnostic and Statistical Manual of Mental Disorders, Fifth Edition (DSM-5), age between 18 and 45 years, and no history of LAI treatment. The assessed period was from January 2010 to December 2019.

The data included the age of onset of the disease, the duration of the disease, the number of relapses, the response to antipsychotic treatment, the adherence to treatment, the number of family members with whom the patient lives and the available formulations of the antipsychotic on which the patient was discharged. Clinical interviews, health and psychiatric records and chart reviews were used to collect participants' data. The scale we used in this research in order to assess adherence was Kemp's 7-point scale. Three board-certified psychiatrists used the ROLIN index separately to check inter-rater reliability. The study was approved by the local ethics committee.

### Participants

During the 10-year research period, 1,116 different patients with schizophrenia were hospitalized. Out of these, 477 (42.7%) met the age eligibility criteria (18 to 45 years old). A total of 225 patients out of 477 (47.1%) with the mean age of 37.8 years (SD ± 5.9) had never had treatment with LAIs and was included in final statistics.

### Rating Opportunity for Long-Acting Injectable Antipsychotic Initiation Index

It is estimated that 50% of patients suffering from chronic illness are not taking medication as prescribed after six months (26). This phenomenon, part of the human nature, is not only present in mental illnesses but and also in chronic diseases such as diabetes, hypertension, cerebrovascular diseases (27). The rate of non-adherence with antipsychotics in schizophrenia varies between studies, reflecting differences in the populations studied and the methodology used in terms of the definition and measurement of adherence and the period of time over which it is assessed (11, 28).

According to our experience correlated with the literature on non-adherence topic (11) we proposed index's items derived from the most important elements involved in the outcome of schizophrenia: age, duration of illness, number of relapses, antipsychotic treatment response, family support and adherence.

- Age. The patient's age is a key factor in the subsequent evolution. Young patients may still have many neurocognitive resources needed for treatment response, remission and recovery (29).

Recent studies have shown that patients in the early stages of the disease can more easily accept this type of treatment (30). Thus, for the age of 18 to 25 years we scored 5 points, for the age of 26 to 35 years we scored 3 points, and for those aged between 36 and 45 years, we scored 1 point.

- Duration of illness. Studies show that neuropathological changes occur in the first years of the disease (31). Thus, the first 2–5 years can be considered of major importance for the patient. We rated 5 points for patients with disease duration of 2 to 5 years, 3 points for duration of 6 to 10 years and 1 point for disease duration over 10 years.
- Relapses. Relapses and hospitalizations in the early years are unfavorable prognostic factors; they predict an evolution toward cognitive decline and chronicity (32, 33). Therefore, experts recommend an early initiation of LAI, after the first relapses caused by non-adherence. We rated 5 points for at least 3 relapses, 3 points for 2 relapses and 1 point for 1 relapse.
- Response to oral antipsychotic. Many young people with schizophrenia respond well to the first trials with antipsychotics, a significant percentage even achieve remission (34). Individual responsiveness and the severity of the pathology are criteria that influence the choice of antipsychotic and the doses. The therapeutic response decreases significantly with the increase in the number of relapses (35). We rated 5 points for complete therapeutic response/remission, 3 points for partial response (residual symptoms at effective doses of oral antipsychotic) and 1 point for lack of response and need for clozapine.
- Family support. Family support is as important as the other elements of therapeutic management (36). In patients with adherence problems, the lack of family members involved in the therapeutic process is considered a predictor of therapeutic abandonment, even when initiating depot formulas. The presence of 2 or more family members close to the patient was rated with 5 points, presence of a single member was scored 3 points and situations where the patient is alone being scored with 1 point.
- Antipsychotic existing formulation. If the patient received an OAP that also has a LAI formulation (aripiprazole, olanzapine, risperidone, and paliperidone) we scored 5 points, if there was only an oral formulation available (amisulpride and quetiapine) we scored 3 points and if the patient was on clozapine 1 point.
- Treatment adherence. Assessing adherence to treatment is a complex and subjective approach. Many methods have been proposed, none of which is perfect. Generally, the schizophrenia patient can be considered as adherent, partially adherent or non-adherent (13, 37). In the patient's file, upon admission, it is mentioned if the patient is adherent, partially adherent or non-adherent. The adherence was evaluated using Kemp's 7-point scale (38). For all variants listed, we rated 1 point for good adherence, 3 points for partial adherence and 5 points for poor adherence.

### Scoring, Interpretation and Recommendations

All 7 items of the index are of equal importance in the management of the schizophrenia patient when LAI initiation is taken into account. The final score, obtained from summing all 7 individual items scores, could range from a minimum of 7 points to a maximum of 35 points, placing the patient in one of the following categories:

- 25–35 points = strong indication for LAI initiation. This score indicates the need for a preventive action. It means that the patient has the premises (age, support, and therapeutic response) and the highest chances of total functional recovery. LAI should be initiated as soon as possible to prevent a new relapse or hospitalization.
- 15–23 points = moderate indication for LAI initiation. This score indicates the need for a better functionality action. It should be interpreted as a patient category that can benefit from LAI for achieving significant improvement in functioning and therefore a better social and professional integration.
- 07–13 points = low indication for LAI initiation. This score indicates the need for a better autonomy action. It means that long-acting treatment could increase the patient's autonomy especially in the cases of patients with low support, low income or homeless.

### Statistical Analysis

For statistical analysis of the data, we used the Statistical Package for Social Sciences (SPSS Inc., Chicago, Illinois, USA) software for Windows (version 21). Demographic, clinical, and treatment variables were compared using Chi Square Tests (categorical variables) or ANOVAs (continuous variables). The sample size was calculated using G-Power analysis. The normal distribution of value was calculated using Kolmogorov-Smirnov test. Interrater reliability was measured using Cohen's kappa statistics. All statistics were two-tailed.

## Results

Patients characteristics of the final sample are presented in Table 2.

**Table 2:** Patients characteristics.

**Parameters**	***N* = 225**
Male gender (n; %)	120; 53.3%
Age (mean, SD)	36.7; ± 5.6
Age group	
− 18–25 years (n; %)	3; 1.4%
− 26–35 years (n; %)	68; 30.2 %
− 36–45 years (n; %)	154; 68.4 %
Duration of illness (mean, SD)	12.4; ± 14.1
Duration of illness group	
− 2–5 years (n; %)	51; 22.7 %
− 6–10 years (n; %)	42; 18.6 %
- >10 years (n; %)	132; 58.7 %
Relapses	
− 2 or more 2 relapses (n; %)	202; 89.8 %
− 1 relapse (n; %)	12; 5.3 %
- No relapse (n; %)	11; 4.9 %
Family support	
− 2 members or more (n; %)	84; 37.3 %
− 1 member (n; %)	92; 40.9 %
- No member (n; %)	49; 21.8 %
Response to oral AP	
- Good response (n; %)	129; 57.3 %
- Partial response (n; %)	93; 41.3 %
- No response (n; %)	3; 1,4 %
Adherence	
- Good (n, %)	31; 13.8 %
- Partial (n, %)	21; 9.3 %
- Non-adherence (n, %)	173; 76.9 %
No of antipsychotics used life time	
− 1 or 2 (n; %)	80; 35.5%
− 3 or 4 (n; %)	95; 42.2%
− 5 or more (n; %)	50; 22.3%

**Figure 1:**
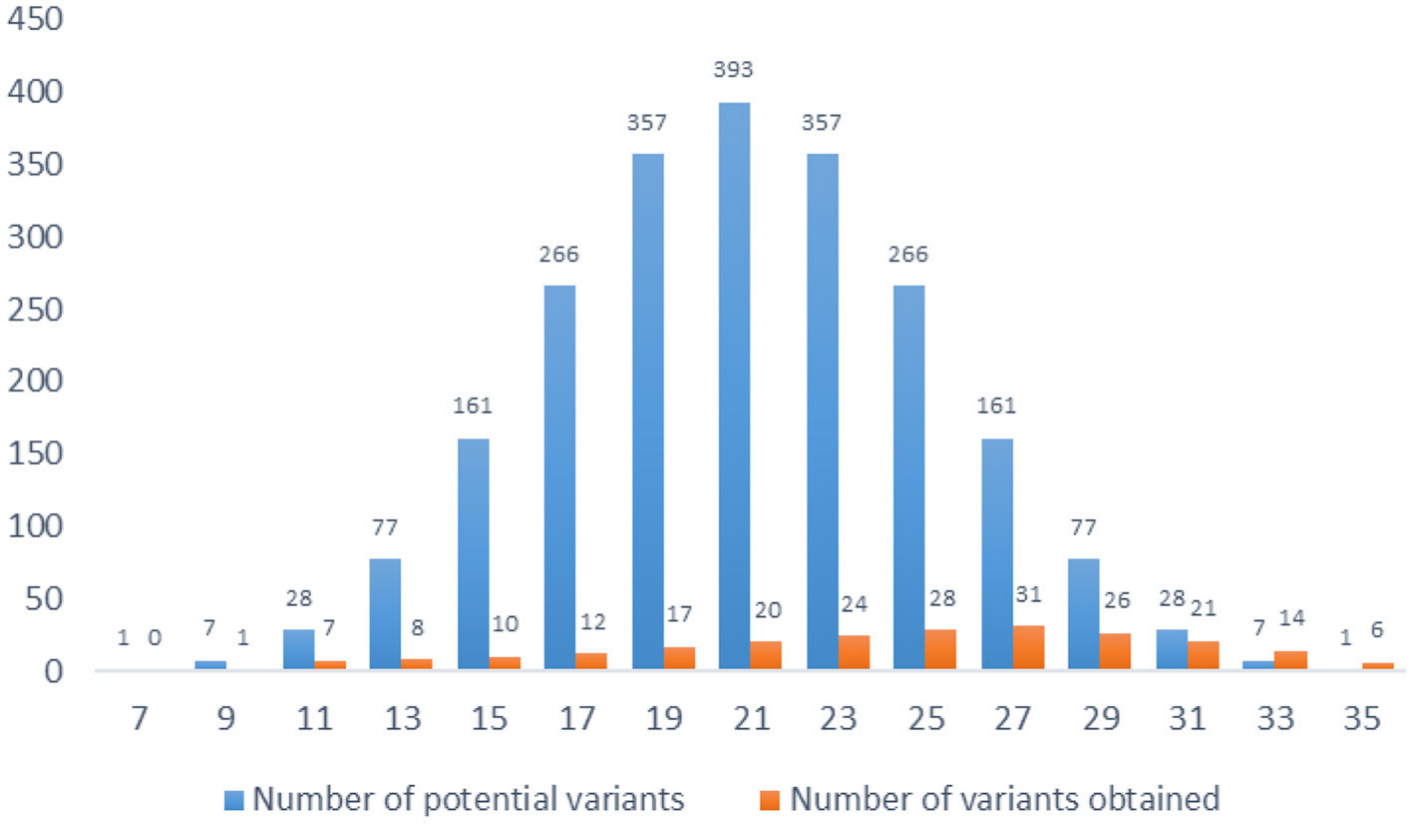
The distribution of ROLIN score.

The total score of the index is calculated by summing the 7 items' scores. There are 15 possible score variants, ranging from 7 to 35. All scores are odd, and some score values appear more frequent than others. In total there are 2,187 (3^7^) variants (Figure 1).

The normal distribution of values was assessed with Kolmogorov-Smirnov test (95% CI for the mean = 9.5781 to 20.4219; 95% CI for the median = 6.5920 to 24.8161; *SD* = 9.7907; Coefficient of Skewness = 0.0743; Coefficient of Kurtosis = −1.1377). The results show a shift to the right because subjects are more likely to get higher results than lower results.

The patients' evaluation with ROLIN showed that 126 patients (56%) had a strong indication for initiating LAI and 88 (39.11%) had a moderate indication. Five patients, discharged on clozapine, without previous long-acting treatment, were considered as having a low indication.

Most patients were young, 57 patients (25.3%) aged in the critical period of the disease (3rd decade of life, between 20 and 30 years) (39). A significant number of patients in this age group had a maximum score in at least four items, indicating the need for LAI initiation. Two hundred and three patients (90.2%) had more than 2 relapses. One hundred and five patients (46.7%) lived with at least one family member. Sixty eight percent of patients were non-adherent, which confirms that 2/3 of patients with schizophrenia are not treatment compliant (Table 3).

**Table 3:** The scale items.

**Items**	**Score**		
	**5 points**	**3 points**	**1 point**
Age (n; years; %)	20; 8.9%	71; 31.5%	134; 59.6 %
Duration of illness (n; years; %)	35; 15.6 %	84; 37.3 %	106; 47.1%
Relapses (n; %)	203; 90.2 %	12; 5.3 %	10; 4.5 %
Response to OAPs (n; %)	140; 62.2 %	78; 34.7 %	7; 3.1 %
Family support (n; %)	84; 37.3 %	105; 46.7 %	36; 16 %
Antipsychotic formulation (n; %)	177; 78.7%	43; 19.1%	5; 2.2%
Adherence (n; %)	154; 68.4 %	55; 24.5 %	16; 7.1 %

The results of our research showed that 177 patients (78.7%) were recommended at discharge an OAP that also had a long-acting formulation. A well-known fact in Romania is the clinicians' affinity for olanzapine, an antipsychotic described as being efficient, having low price, and affordable even for the uninsured patients, largely available and having a long-acting formulation. Olanzapine long-acting formulation has the disadvantage of requiring post-injection monitoring for a period of 3 h, which makes it difficult to initiate (a fact that was even more obvious in the current COVID-19 pandemic) (40). The antipsychotics recommended at discharge are presented in Table 4.

**Table 4:** Antipsychotic used at discharge.

**Oral antipsychotics**			**Concomitant medication**					
**OAPs**	**n; %**	**Dose (mean, SD)**	**MS (n; %)**	**BZD (n; %)**	**ACh (n; %)**	**HYP (n; %)**	**AD (n; %)**	**2ndAP (n; %)**
OLZ	124; 55.1%	15.3 mg; ± 4.7	102; 45.3%	116; 51.5%	28; 12.4%	20; 20.9%	4; 1.7%	23; 10.2%
QUE	25; 11.1%	550 mg; ± 151	17; 68%	15; 60%	6; 24%	-	-	3; 12%
CLO	5; 2.2%	300 mg ± 112	-	-	-	-	-	-
RIS	28; 12.4%	3.2 mg; ± 0.91	8; 28.6%	15; 53.6%	12; 42.9%	6; 21.4%	2; 7.1%	-
PAL	11; 4.9%	7.5 mg; ± 1.73	4; 36.4%	5; 45.5%	-	3; 27.3%	-	-
AMI	13; 5.8%	514 mg; ± 195	4; 30.7%	6; 46.1%	1; 7.7%	2; 15.4%	-	-
ARI	9; 4%	18 mg; ± 8.3	4; 44.4%	3; 33.3%	-	3; 33.3%	-	-
HAL	10; 4.4%	7,7 mg; ± 2.9	6; 60%	8; 80%	5; 50%	-	-	-

*OAPs, oral antipsychotics; OLZ, olanzapine; QUE, quetiapine; CLO, clozapine; RIS, risperidone; PAL, paliperidone; AMI, amisulpride; ARI, aripiprazole; HAL, haloperidol; MS, mood stabilizer; BZD, benzodiazepine; ACh, anticholinergic; HYP, hypnotic; AD, antidepressant; 2nd AP, concomitant use of a 2nd antipsychotic*.

The results of our calculations show that our reliability for items assessing ROLIN items is good (Table 5). All 7 items obtained adequate coefficient scores (0.82–1) meaning near perfect agreement.

**Table 5:** Interrater reliability for ROLIN items.

**ROLIN item**	**Kappa**	**Acceptability**
Age	1	Adequate
Duration of illness	0.86	Adequate
Relapse	0.92	Adequate
Response to oral antipsychotic	0.82	Adequate
Patient social support	0.96	Adequate
Antipsychotic formulation	1	Adequate
Treatment adherence	0.88	Adequate

*ROLIN, rating opportunity for LAI initiation index. All coefficients are within the adequate range (0.82–1.00)*.

## Discussion

We have described the development and initial standardization of the 7-item index as an instrument for measuring the opportunity for LAI initiation in schizophrenia. As far as we know, this is the first index created to encourage the initiation of LAI in schizophrenia, providing evidence and supporting the early initiation of this treatment. Statistical analysis showed a normal distribution of values which is a very important thing that validates the index (skewness = 0.0743; kurtosis = −1.1377).

Although many studies indicate that the use of LAIs may have the potential to prevent relapses and hospitalizations compared to oral antipsychotics (41), and although second-generation LAI formulations are widely available, it has been estimated that only 10–20% of eligible patients are actually prescribed LAIs (42). The results of our research show that a significant number of patients who had an indication for LAI were discharged on OAPs.

One explanation for why these agents are under-initiated may be the current lack of clear and practical guidelines on how and to whom we should initiate treatment with these agents. Studies show that initiation is common in severe patients, with involuntary hospitalizations, with evidence of non-adherence to treatment (43). This could lead to a decline in LAI confidence. Fear of more severe side effects may be another explanation, although meta-analyzes show that there are no differences between LAIs and OAPs (44). Despite availability of all LAIs formulation in Romania, free of charge for insured patients diagnosed with schizophrenia, a significant percentage of patients with a strong indication for LAIs (64%) were still discharged on OAPs.

We must emphasize that the presence of a lower score should not be a barrier in initiating LAI treatment. Initiation of LAI in these patients, if followed by all measures to ensure adherence (government programs, institutional support, etc.) can lead to reduced hospitalizations, lower direct and indirect costs and an acceptable degree of patient autonomy.

This index can also enhance the patient's collaboration and the families' engagement in the therapeutic plan, because presenting them the need for initiation score could build a stronger confidence in the clinician's judgment. With minimal time investment, patients and their families could become sufficiently informed in order to accept LAI initiation. It could also help psychiatrists that work only in the outpatient facilities or in private practices to initiate LAI, because this type of treatment is usually initiated in the hospital.

### Limitations

Like any instrument, the ROLIN has several limitations. One limitation is the relative small number of cases. Another limitation could be the application of the LAI initiation index to pregnant patients, in which case initiation still raises widespread debate (45) or in the first episode schizophrenia. The present study design did not specifically address effectiveness of the proposed index.

The index must be interpreted with recommendations. In the case of patients with no relapse, there still remains the possibility of therapeutic abandonment, so initiation is a preventive measure. In treatment-resistant schizophrenia, the clinician must assess if it is really a case of treatment resistance or it is rather treatment non-adherence. In the case of a false treatment resistance situation, we recommend LAI initiation. Based on our strong experience in patients on clozapine for treatment-resistance or for aggressive behavior, a switch to LAI is not recommended. In cases of severe adverse events this switch must be done with caution.

## Conclusion

The validity of the ROLIN should be upheld by future studies with independent investigators: Its use might be expected to promote uniformity and reliability in research findings.

## Data Availability Statement

Data is available from the corresponding author upon reasonable request.

## Author Contributions

PI and AT: conception, design of the research, and drafting the manuscript. P-SP: acquisition of data and statistical analysis. PI: analysis and interpretation of data. All authors have read and approved the final manuscript.

## Funding

The author(s) declare that no financial support was received for the research, authorship, and/or publication of this article.

## Correction Note

A correction has been made to this article. Details can be found at 10.3389/fpsyt.2025.1718142.

## Conflict of Interest

The authors declare that the research was conducted in the absence of any commercial or financial relationships that could be construed as a potential conflict of interest.

## Publisher's Note

All claims expressed in this article are solely those of the authors and do not necessarily represent those of their affiliated organizations, or those of the publisher, the editors and the reviewers. Any product that may be evaluated in this article, or claim that may be made by its manufacturer, is not guaranteed or endorsed by the publisher.

## References

[1] Kahn RS Sommer IE Murray RM Meyer-Lindenberg A Weinberger DR Cannon TD. Schizophrenia. Nat Rev Dis Primers. (2015) 1:15067. 10.1038/nrdp.2015.67 27189524

[2] Switaj P Anczewska M Chrostek A Sabariego C Cieza A Bickenbach J. Disability and schizophrenia: a systematic review of experienced psychosocial difficulties. BMC Psychiatry. (2012) 12:193. 10.1186/1471-244X-12-193 23137171 PMC3539983

[3] Correll CU Solmi M Veronese N Bortolato B Rosson S Santonastaso P. Prevalence, incidence and mortality from cardiovascular disease in patients with pooled and specific severe mental illness: a large-scale meta-analysis of 3,211,768 patients and 113,383,368 controls. World Psychiatry. (2017) 16:163–80. 10.1002/wps.20420 28498599 PMC5428179

[4] Mitchell AJ Vancampfort D Sweers K van Winkel R Yu W De Hert M. Prevalence of metabolic syndrome and metabolic abnormalities in schizophrenia and related disorders–a systematic review and meta-analysis. Schizophr Bull. (2013) 39:306–18. 10.1093/schbul/sbr148 22207632 PMC3576174

[5] Ifteni P Correll CU Burtea V Kane JM Manu P. Sudden unexpected death in schizophrenia: autopsy findings in psychiatric inpatients. Schizophr Res. (2014) 155:72–6. 10.1016/j.schres.2014.03.011 24704220

[6] Hjorthøj C Stürup AE McGrath JJ Nordentoft M. Years of potential life lost and life expectancy in schizophrenia: a systematic review and meta-analysis. Lancet Psychiatry. (2017) 4:295–301. 10.1016/S2215-0366(17)30078-0 28237639

[7] Charlson FJ Ferrari AJ Santomauro DF Diminic S Stockings E Scott JG. Global epidemiology and burden of schizophrenia: findings from the global burden of disease study 2016. Schizophr Bull. (2018) 44:1195–203. 10.1093/schbul/sby058 29762765 PMC6192504

[8] Lysaker PH Pattison ML Leonhardt BL Phelps S Vohs JL. Insight in schizophrenia spectrum disorders: relationship with behavior, mood and perceived quality of life, underlying causes and emerging treatments. World Psychiatry. (2018) 17:12–23. 10.1002/wps.20508 29352540 PMC5775127

[9] Shafrin J Silverstein AR MacEwan JP Lakdawalla DN Hatch A Forma FM. Using information on patient adherence to antipsychotic medication to understand their adherence to other medications. P T. (2019) 44:350–7. 31160870 PMC6534179

[10] Johnson S Sathyaseelan M Charles H Jeyaseelan V Jacob KS. Insight, psychopathology, explanatory models and outcome of schizophrenia in India: a prospective 5-year cohort study. BMC Psychiatry. (2012) 12:159. 10.1186/1471-244X-12-159 23013057 PMC3514157

[11] Lacro JP Dunn LB Dolder CR Leckband SG Jeste DV. Prevalence of and risk factors for medication nonadherence in patients with schizophrenia: a comprehensive review of recent literature. J Clin Psychiatry. (2002) 63:892–909. 10.4088/JCP.v63n1007 12416599

[12] Tiihonen J Mittendorfer-Rutz E Majak M Mehtälä J Hoti F Jedenius E. Real-world effectiveness of antipsychotic treatments in a nationwide cohort of 29 823 patients with schizophrenia. JAMA Psychiatry. (2017) 74:686–93. 10.1001/jamapsychiatry.2017.1322 28593216 PMC5710250

[13] Kane JM Correll CU. Optimizing treatment choices to improve adherence and outcomes in schizophrenia. J Clin Psychiatry. (2019) 80:IN18031AH1C. 10.4088/JCP.IN18031AH1C 31536686

[14] Taipale H Mittendorfer-Rutz E Alexanderson K Majak M Mehtälä J Hoti F. Antipsychotics and mortality in a nationwide cohort of 29,823 patients with schizophrenia. Schizophr Res. (2018) 197:274–80. 10.1016/j.schres.2017.12.010 29274734

[15] Nasrallah HA. Triple advantages of injectable long acting second generation antipsychotics: Relapse prevention, neuroprotection, and lower mortality. Schizophr Res. (2018) 197:69–70. 10.1016/j.schres.2018.02.004 29506767

[16] Taylor DM Velaga S Werneke U. Reducing the stigma of long acting injectable antipsychotics - current concepts and future developments. Nord J Psychiatry. (2018) 72(Suppl. 1):S36–9. 10.1080/08039488.2018.1525638 30688170

[17] Law MR Soumerai SB Ross-Degnan D Adams AS. A longitudinal study of medication nonadherence and hospitalization risk in schizophrenia. J Clin Psychiatry. (2008) 69:47–53. 10.4088/JCP.v69n0107 18312037

[18] Sajatovic M Ross R Legacy SN Byerly M Kane JM DiBiasi F. Initiating/maintaining long-acting injectable antipsychotics in schizophrenia/schizoaffective or bipolar disorder - expert consensus survey part 2. Neuropsychiatr Dis Treat. (2018) 14:1475–92. 10.2147/NDT.S167485 29922063 PMC5997122

[19] Kishimoto T Sanghani S Russ MJ Marsh AN Morris J Basu S. Indications for and use of long-acting injectable antipsychotics: consideration from an inpatient setting. Int Clin Psychopharmacol. (2017) 32:161–8. 10.1097/YIC.0000000000000165 28181959 PMC5808869

[20] Marcus SC Zummo J Pettit AR Stoddard J Doshi JA. Antipsychotic adherence and rehospitalization in schizophrenia patients receiving oral versus long-acting injectable antipsychotics following hospital discharge. J Manag Care Spec Pharm. (2015) 21:754–68. 10.18553/jmcp.2015.21.9.754 26308223 PMC10398026

[21] Keepers GA Fochtmann LJ Anzia JM Benjamin S Lyness JM Mojtabai R. The American Psychiatric Association practice guideline for the treatment of patients with schizophrenia. Am J Psychiatry. (2020) 177:868–72. 10.1176/appi.ajp.2020.177901 32867516

[22] Llorca PM Abbar M Courtet P Guillaume S Lancrenon S Samalin L. Guidelines for the use and management of long-acting injectable antipsychotics in serious mental illness. BMC Psychiatry. (2013) 13:340. 10.1186/1471-244X-13-340 24359031 PMC3898013

[23] American Psychiatric Association. The American Psychiatric Association Practice Guideline for the Treatment of Patients With Schizophrenia. 3rd Edn. Washington, DC: American Psychiatric Association (2021).

[24] Citrome L. Long-acting injectable antipsychotics: what, when, and how. CNS Spectr. (2021) 26:118–29. 10.1017/S1092852921000778 33928884

[25] Correll CU Lauriello J. Long-acting injectable antipsychotics: where do they fit in the treatment plan? J Clin Psychiatry. (2018) 79:AL17017WC1C. 10.4088/JCP.AL17017WC1C 29505193

[26] World Health Organization. Adherence to Long-Term Therapies: Evidence for Action. Geneva: World Health Organization (2003).

[27] Kane JM Kishimoto T Correll CU. Non-adherence to medication in patients with psychotic disorders: epidemiology, contributing factors and management strategies. World Psychiatry. (2013) 12:216–26. 10.1002/wps.20060 24096780 PMC3799245

[28] Byerly M Fisher R Whatley K Holland R Varghese F. A comparison of electronic monitoring vs clinician rating of antipsychotic adherence in outpatients with schizophrenia. Psychiatry Res. (2005) 133:129–33. 10.1016/j.psychres.2004.11.002 15740989

[29] Albert N Bertelsen M Thorup A Petersen L Jeppesen P Le Quack P. Predictors of recovery from psychosis analyses of clinical and social factors associated with recovery among patients with first-episode psychosis after 5 years. Schizophr Res. (2011) 125:257–66. 10.1016/j.schres.2010.10.013 21056926

[30] Kane JM Schooler NR Marcy P Achtyes ED Correll CU Robinson DG. Patients with early-phase schizophrenia will accept treatment with sustained-release medication (long-acting injectable antipsychotics): results from the recruitment phase of the PRELAPSE trial. J Clin Psychiatry. (2019) 80:18m12546. 10.4088/JCP.18m12546 31050233

[31] Kane JM Schooler NR Marcy P Correll CU Achtyes ED Gibbons RD. Effect of long-acting injectable antipsychotics vs usual care on time to first hospitalization in early-phase schizophrenia: a randomized clinical trial. JAMA Psychiatry. (2020) 77:1–8. 10.1001/jamapsychiatry.2020.2076 32667636 PMC7364341

[32] Chi MH Hsiao CY Chen KC Lee LT Tsai HC Hui Lee I. The readmission rate and medical cost of patients with schizophrenia after first hospitalization - a 10-year follow-up population-based study. Schizophr Res. (2016) 170:184–90. 10.1016/j.schres.2015.11.025 26678982

[33] Tiihonen J Taipale H Mehtälä J Vattulainen P Correll CU Tanskanen A. Association of antipsychotic polypharmacy vs monotherapy with psychiatric rehospitalization among adults with schizophrenia. JAMA Psychiatry. (2019) 76:499–507. 10.1001/jamapsychiatry.2018.4320 30785608 PMC6495354

[34] Ventura J Subotnik KL Guzik LH Hellemann GS Gitlin MJ Wood RC. Remission and recovery during the first outpatient year of the early course of schizophrenia. Schizophr Res. (2011) 132:18–23. 10.1016/j.schres.2011.06.025 21764563 PMC3172347

[35] Takeuchi H Siu C Remington G Fervaha G Zipursky RB Foussias G. Does relapse contribute to treatment resistance? Antipsychotic response in first- vs second-episode schizophrenia. Neuropsychopharmacology. (2019) 44:1036–42. 10.1038/s41386-018-0278-3 30514883 PMC6462044

[36] McFarlane WR. Family interventions for schizophrenia and the psychoses: a review. Fam Process. (2016) 55:460–82. 10.1111/famp.12235 27411376

[37] Smith RL Tveito M Kyllesø L Jukic MM Ingelman-Sundberg M Andreassen OA. Rates of complete nonadherence among atypical antipsychotic drugs: a study using blood samples from 13,217 outpatients with psychotic disorders. Schizophr Res. (2021) 228:590–6. 10.1016/j.schres.2020.11.025 33221147

[38] Kemp R Hayward P Applewhaite G Everitt B David A. Compliance therapy in psychotic patients: randomised controlled trial. BMJ. (1996) 312:345–9. 10.1136/bmj.312.7027.345 8611831 PMC2350295

[39] Lieberman JA Perkins D Belger A Chakos M Jarskog F Boteva K. The early stages of schizophrenia: speculations on pathogenesis, pathophysiology, and therapeutic approaches. Biol Psychiatry. (2001) 50:884–97. 10.1016/S0006-3223(01)01303-8 11743943

[40] Ifteni P Dima L Teodorescu A. Long-acting injectable antipsychotics treatment during COVID-19 pandemic - a new challenge. Schizophr Res. (2020) 220:265–6. 10.1016/j.schres.2020.04.030 32349886 PMC7185008

[41] Kishimoto T Nitta M Borenstein M Kane JM Correll CU. Long-acting injectable versus oral antipsychotics in schizophrenia: a systematic review and meta-analysis of mirror-image studies. J Clin Psychiatry. (2013) 74:957–65. 10.4088/JCP.13r08440 24229745

[42] Patel MX Bent-Ennakhil N Sapin C di Nicola S Loze JY Nylander AG. Attitudes of European physicians towards the use of long-acting injectable antipsychotics. BMC Psychiatry. (2020) 20:123. 10.1186/s12888-020-02530-2 32169077 PMC7071632

[43] Lin CH Chen FC Chan HY Hsu CC. Time to rehospitalization in patients with schizophrenia receiving long-acting injectable antipsychotics or oral antipsychotics. Int J Neuropsychopharmacol. (2019) 22:541–7. 10.1093/ijnp/pyz035 31260538 PMC6754732

[44] Misawa F Kishimoto T Hagi K Kane JM Correll CU. Safety and tolerability of long-acting injectable versus oral antipsychotics: a meta-analysis of randomized controlled studies comparing the same antipsychotics. Schizophr Res. (2016) 176:220–30. 10.1016/j.schres.2016.07.018 27499361

[45] Teodorescu A Ifteni P Moga MA Burtea V Bigiu N. Dilemma of treating schizophrenia during pregnancy: a case series and a review of literature. BMC Psychiatry. (2017) 17:311. 10.1186/s12888-017-1475-z 28851326 PMC5576383

