# Correction: Rating opportunity for long-acting injectable antipsychotic initiation index (ROLIN)

**DOI:** 10.3389/fpsyt.2025.1718142

**Published:** 2025-10-31

**Authors:** Petru Ifteni, Paula-Simina Petric, Andreea Teodorescu

**Affiliations:** Faculty of Medicine, Transilvania University of Braşov, Braşov, Romania

**Keywords:** schizophrenia, antipsychotic, treatment, relapse, outcome, long-acting injectable

The **Data Availability Statement** was erroneously given as “The raw data supporting the conclusions of this article will be made available by the authors, without undue reservation.”

The correct **Data Availability Statement** is “Data is available from the corresponding author upon reasonable request.”

An incorrect **Funding** statement was included in the published article:

“This work was supported by Beijing Municipal Natural Science Foundation (7202189), Project of National Clinical Research Center for Geriatric Disease (NCRCG-PLAGH- 2019024), The National Key Research Program of China (2018YFC0116305), The National Natural Science Foundation of China (grant nos. 91939303 and 81820108019), and Major Program of Central Health Committee (2020ZD05).”

The correct **Funding** statement reads:

“The author(s) declare that no financial support was received for the research, authorship, and/or publication of this article.”

The original version of this article has been updated.

